# Structure and Dynamics of Meprin β in Complex with a Hydroxamate-Based Inhibitor

**DOI:** 10.3390/ijms22115651

**Published:** 2021-05-26

**Authors:** Miriam Linnert, Claudia Fritz, Christian Jäger, Dagmar Schlenzig, Daniel Ramsbeck, Martin Kleinschmidt, Michael Wermann, Hans-Ulrich Demuth, Christoph Parthier, Stephan Schilling

**Affiliations:** 1 Fraunhofer Institute for Cell Therapy and Immunology, Department of Drug Design and Target Validation, Weinbergweg 22, 06120 Halle (Saale), Germany; claudia.spahn@izi.fraunhofer.de (C.F.); dagmar.schlenzig@izi.fraunhofer.de (D.S.); daniel.ramsbeck@izi.fraunhofer.de (D.R.); martin.kleinschmidt@izi.fraunhofer.de (M.K.); michael.wermann@izi.fraunhofer.de (M.W.); hans-ulrich.demuth@t-online.de (H.-U.D.); 2 Vivoryon Therapeutics N. V., Weinbergweg 22, 06120 Halle (Saale), Germany; christian.jaeger@izi-extern.fraunhofer.de; 3 Faculty of Applied Biosciences and Process Engineering, Anhalt University of Applied Sciences, Bernburger Street 55, 06366 Köthen, Germany; 4 Institute of Biochemistry and Biotechnology, Charles-Tanford-Proteinzentrum, Martin-Luther-University Halle-Wittenberg, Kurt-Mothes-Street 3a, 06120 Halle (Saale), Germany; christoph.parthier@biochemtech.uni-halle.de

**Keywords:** Meprin B, Meprin beta, metalloproteinase, astacin, hydroxamate, SAR (structure activity relationship), MWT-S-270

## Abstract

The astacin protease Meprin β represents an emerging target for drug development due to its potential involvement in disorders such as acute and chronic kidney injury and fibrosis. Here, we elaborate on the structural basis of inhibition by a specific Meprin β inhibitor. Our analysis of the crystal structure suggests different binding modes of the inhibitor to the active site. This flexibility is caused, at least in part, by movement of the C-terminal region of the protease domain (CTD). The CTD movement narrows the active site cleft upon inhibitor binding. Compared with other astacin proteases, among these the highly homologous isoenzyme Meprin α, differences in the subsites account for the unique selectivity of the inhibitor. Although the inhibitor shows substantial flexibility in orientation within the active site, the structural data as well as binding analyses, including molecular dynamics simulations, support a contribution of electrostatic interactions, presumably by arginine residues, to binding and specificity. Collectively, the results presented here and previously support an induced fit and substantial movement of the CTD upon ligand binding and, possibly, during catalysis. To the best of our knowledge, we here present the first structure of a Meprin β holoenzyme containing a zinc ion and a specific inhibitor bound to the active site. The structural data will guide rational drug design and the discovery of highly potent Meprin inhibitors.

## 1. Introduction

Meprins (Meprin α and β) are multidomain, Zn-dependent proteases. They belong to the astacin family and metzincin superfamily of endo-proteinases. Originally discovered in the early 1980s in human intestine and mouse kidney [1,2,3], Meprins have only been described in vertebrate tissue such as human skin, leukocytes and various cancer cells. They are involved in a broad range of proteolytic processes, among these connective tissue homeostasis and immunological and intestinal barrier function [4,5,6]. Meprins are key players in the processing of procollagen I and III and are potentially involved in pathological conditions such as fibrosis or keloids. Hence, the development of selective and potent inhibitors represents a vital approach for treatment of fibrosis, nephritis and neurodegeneration [7].

From a structural perspective, meprins are intermolecular disulfide-bridged homo-dimeric or heterodimeric complexes of the evolutionary related multidomain subunits, Meprin α and Meprin β [8,9]. Meprin A (EC 3.4.24.18) is either composed of αβ-heterodimers, which tends to form tetramers (α_2_β_2_, α_3_β_1_), or composed of αα-homodimers with up to 100 subunits, which form ring-, circle-, spiral- and tube-like non-covalent congregated complexes. Meprin B (EC 3.4.24.63) is only known as a homodimer of β subunits [10,11]. Each α and β subunit contains an N-terminal signal peptide for translocation into the secretory pathway and a pro-peptide, which is removed from the zymogen by trypsin-like proteinases upon activation [12]. The pro-peptide is followed by an astacin-like protease domain, a MAM domain (Meprin A5 protein tyrosine phosphatase µ), and a TRAF domain (tumor-necrosis-factor-receptor-associated factor). The MAM and TRAF domains are involved in protein–protein interactions and signal transduction [13,14,15]. The cysteine residues that form a dimer-connecting intermolecular disulfide bridge are located within the MAM domain. An EGF-like domain (epidermal growth factor like), a transmembrane domain and a cytosolic tail form the C-terminal region of meprins [3,16]. The major structural difference between Meprin α and β represents an inserted domain containing a furin cleavage site, which is located N-terminal of the EGF-like domain in Meprin α. Furin cleavage of Meprin α leads to the release of complexes composed only of Meprin α into the extracellular space, whereas Meprin β-containing complexes are still located at the cell membrane [17,18].

The first structure of the family prototypic astacin from freshwater crayfish *Astacus astacus* was solved by X-ray crystallography in 1992 [19,20]. The crystal structure of the multidomain Meprin β (Meprin B) was solved 20 years later [21]. The protease domains of the enzymes show a remarkably high structural similarity. The protease domains fold into an N-terminal “upper” (NTS) and a C-terminal “lower” subdomain (CTS). Both subdomains are separated by a deep and narrow active site cleft, which harbors a catalytic zinc ion at its base. As with all proteases from the metzincin superfamily, astacins possess the conserved zinc-binding motif HExxHxxGxxH. This is followed by an astacin-characteristic glutamate residue C-terminal of the third histidine and the so-called Met-turn [22,23,24]. This is a five-residues-containing loop with a strictly conserved methionine at the third and a tyrosin at the fifth position. In activated astacins, the catalytic zinc ion is coordinated by the three histidins from the zinc binding motif and a water molecule. In the prototypic astacin protease from *Astacus astacus,* the hydroxyl group of the afore mentioned tyrosine from the Met-turn coordinates the zinc at a fifth side, whereby in other astacins, including the meprins, the coordination of the zinc ion by this tyrosine is not fully clear [21,25]. The substrates of the astacin proteinases bind while sprawled over the active site cleft. Binding involves at least four amino acids each on the N-terminal non-prime (P) and the C-terminal prime site (P’) of the scissile peptide bond [26,27]. Meprin β shows a striking preference for negatively charged amino acids proximal to the scissile bond [28,29].

On the basis of the metalloproteinase inhibitor N-isobutyl-N-(4-methoxy- phenylsulfonyl)-glycyl hydroxamic acid (NNGH) [30], we reported the development of selective and potent synthetic small molecule inhibitors of Meprin α and β [31,32,33] by bio-isosteric replacement of the sulfonamide residue by a tertiary amine. This replacement led to an inhibition of astacin metalloproteinases, but not of other investigated members of the metzincin family. While retaining the hydroxamic acid and tertiary amine scaffold, differentially substituted inhibitors have been developed by elucidating their structure-activity relationships (SAR), resulting in a selective inhibitor of Meprin β with two meta–substituted carboxy-benzyl side chains (MWT-S-270; 3-[[(3-Carboxyphenyl)methyl-[2-(hydroxyamino)-2-oxoethyl]amino]methyl]benzoic acid). It was shown that the acidic substitution is important for the high inhibitory potency against Meprin β over Meprin α or other related proteinases, e.g., MMPs and ADAMs [33]. The SAR and the accompanying in silico docking studies led to the assumption that ionic interactions between the meta-carboxylic acid groups and Arg^184^ in the S1 subsite and/or Arg^238^ in the S1′ subsite might be important for inhibitor binding [33]. To investigate this interaction in detail, we crystallized the catalytically active ectodomain of human Meprin β in complex with the tertiary-amine inhibitor MWT-S-270. To our knowledge, this is the first attempt to crystallize Meprin β in complex with a specific small-molecule inhibitor.

## 2. Results

### 2.1. Preparation of Meprin β^62–595^ (MβΔC) for Crystallization

**Figure 1:**
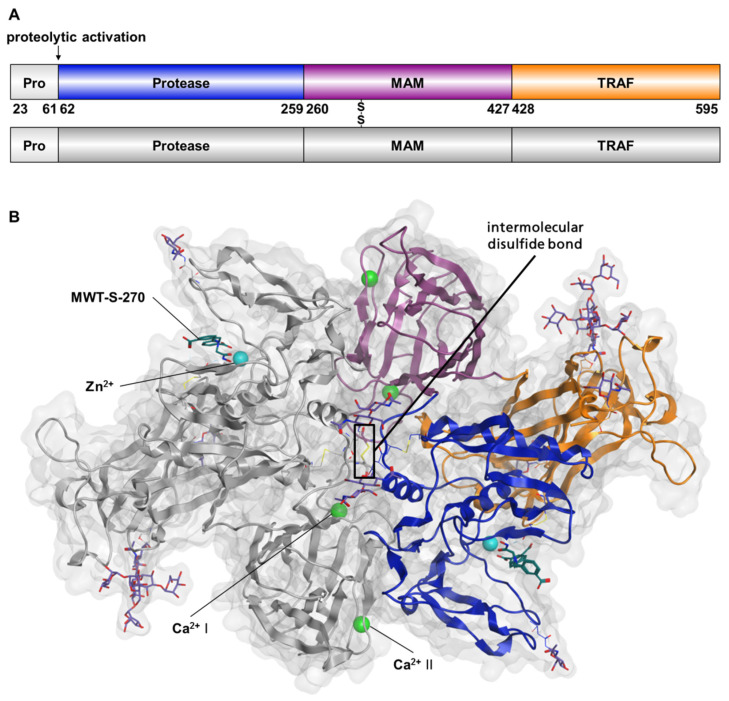
Structure of the Meprin β homodimer (Meprin B). (**A**) Schematic representation of the human Meprin β construct (pMβΔC) isolated from *Pichia pastoris* after heterologous expression. The protein includes the pro-peptide (Thr^23^-Arg^61^, light grey), the protease domain (Asn^62^-Leu^259^; blue), the MAM-domain (Ser^260^-Cys^427^; magenta) and the TRAF-domain (Pro^428^-Gln^595^; orange). The second monomer of the homodimer is shown in plain dark grey. After activation by trypsin the mature protein consists of amino acids Asn^62^-Gln^595^ (MβΔC). Residue numbering according to Arolas et al. [21]. (**B**) Solved structure of the Meprin β^62–595^ (MβΔC) homodimer, connected by an intermolecular disulfide-bridge between Cys^305^ of monomer A and B. The same color code was chosen as in the schematic representation (monomer A colored, monomer B grey). Additionally, the molecular surface is shown in light grey. Protein N-glycosylation sites at asparagine side chains are represented as light blue sticks. Two calcium ions (green sphere), I and II, as well as a zinc ion (cyan sphere) and the inhibitor MWT-S-270 (dark green sticks) in the active site (protease domain) are shown in both monomers.

Meprin β was produced in *Pichia pastoris*, applying an optimized version of a previously described protocol [34]. Compared with the previous approach, we modified the expression construct including the pro-peptide-, catalytic, MAM- and TRAF-domain. The formerly described N-His-Meprin β^23–652^ was shortened resulting in N-His-Meprin β^23–595^ (pMβΔC) (Figure 1A) in order to remove flexible regions. The activation of Pro-Meprin by trypsin resulted in removal of the poly-histidin tag and the pro-peptide (Figure S1). For the purified, truncated variant Meprin β^62–595^ (MβΔC), despite reduced protein yield, identical specific activity was detected when compared with the previous variant Meprin β^62–562^ (not shown).

For reduction of potential heterogeneity, flexible glycosyl chains were removed by deglycosylation. The influence of different detergents and protein concentrations on the deglycosylation and stability of the enzyme has been investigated and analyzed by SDS-PAGE. At high Meprin β^62–595^ (MβΔC) concentrations, protein precipitation was observed during the deglycosylation process. Hence, the concentration of Meprin β^62–595^ (MβΔC) was kept at 0.5 mg/mL for deglycosylation (Figure S2A). In the progress of protein crystallization, a stability test using the concentrated protein was performed at 15 °C for up to 15 days. In absence of an inhibitor, Meprin β^62–595^ (MβΔC) was degraded under these conditions (Figure S2B), obviously due to proteolytic cleavage. To prevent degradation, the non-specific Meprin β inhibitor actinonin or the specific Meprin β inhibitor MWT-S-270 was added to the protein solution in a molar ratio of 1:1.3 and 1:1.2, respectively. By addition of one of these inhibitors, the degradation of Meprin β^62–595^ (MβΔC) was prevented (Figure S2C), supporting auto-proteolytic activity at high enzyme concentration [35]. Therefore, Meprin β^62–595^ (MβΔC) was crystallized only in the presence of MWT-S-270.

### 2.2. Structure of Meprin β^62–595^ (MβΔC) in Complex with MWT-S-270

The mature Meprin β variant Meprin β^62–595^ (MβΔC) crystallized in the presence of MWT-S-270 in two different space groups, the already reported [21] hexagonal space group P6_1_22 (178) (data not shown) and, additionally, under the conditions reported here, in the monoclinic space group C2 (5), with two monomers in the asymmetric unit. Data sets from a monoclinic crystal were collected and analyzed to a resolution of 2.4 Å. After phasing by molecular replacement and iterative cycles of model building and refinement, a final model with an R_work_ = 0.20 and R_free_ = 0.24 was achieved. Data collection and refinement statistics are given in Table 1.

The final model comprises two Meprin β^62–595^ monomers, A and B, containing the amino acids Asn^62^-Thr^594^. Electron density was not observed for the terminal residue Gln^595^. Each monomer spans the catalytic active protease domain (Asn^62^-Leu^259^), the MAM-domain (Ser^260^-Cys^427^) and the TRAF-domain (Pro^428^-Gln^595^) (Figure 1B). Both monomers are covalently linked through an intermolecular disulfide bridge connecting residue Cys^305^ of monomer A and B. This feature could not be observed in the crystal structure described previously [21]. Additionally, electron density was observed for four intramolecular disulfide bonds per monomer, connecting Cys^103^-Cys^255^, Cys^124^-Cys^144^, Cys^265^-Cys^273^ and Cys^340^-Cys^427^.

To facilitate crystallization, the purified Meprin β^62–595^ (MβΔC) was deglycosylated by Endoglycosidase H, which cleaves the 1,4-β-O-glycosidic bond between the first and the second *N*-*acetylglucosamine residue* linked to the asparagine side chain. Apparently, Asn^218^, Asn^445^ and Asn^592^ were deglycosylated by Endo H treatment, whereas Asn^254^ still harbors 2 *N*-*acetylglucosamine residues*. Asn^547^ was not deglycosylated in both monomers under the chosen conditions. Each monomer comprises at least three bound metal ions: two tentative calcium ions and a catalytic zinc ion. One calcium (I) is octahedrally coordinated in both monomers by Asp^418^(bidentate), Asp^298^, Glu^268^, Ser^300^, Ser^266^ and Phe^310^ (Figure S3A,B). The presence of calcium is supported by anomalous scattering, the characteristic metal–protein distances and the octahedral coordination geometry [37]. A second calcium cation (II) is coordinated by the protein ligands Asp^281^ (bidentate), Ser^278^, Ala^283^ and Asp^284^ in both monomers (Figure S3C,D).

**Figure 2:**
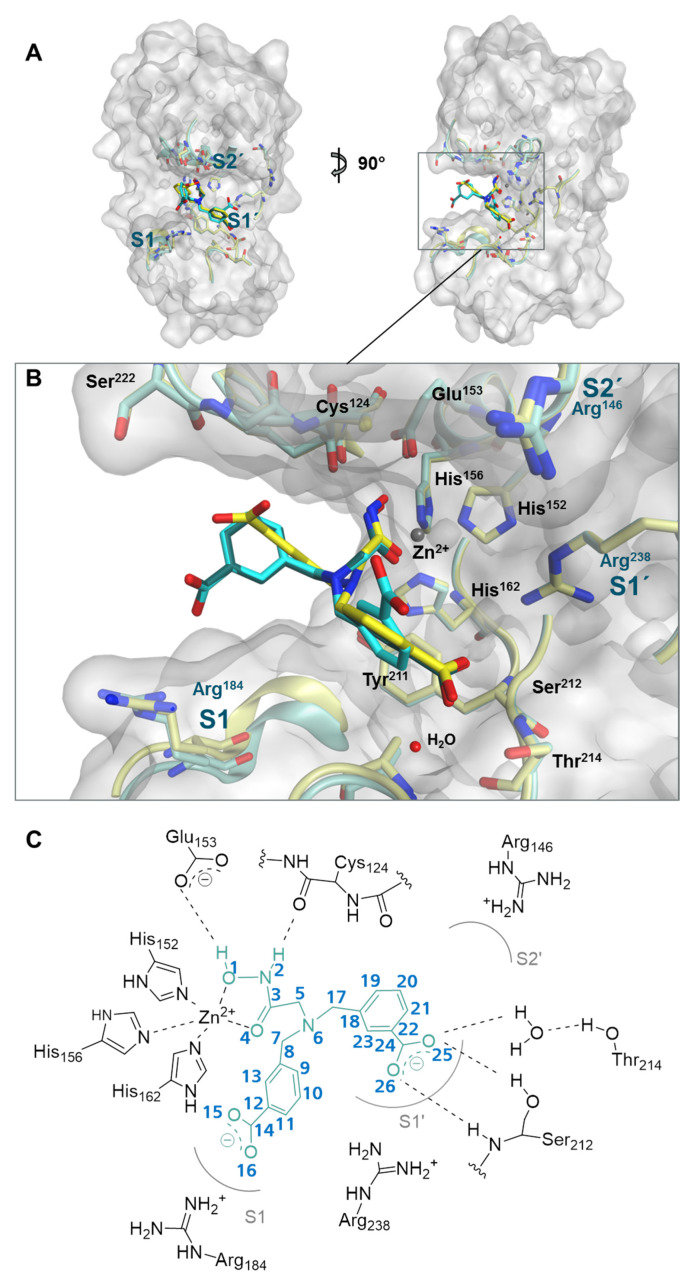
Interaction of MWT-S-270 with the active site of Meprin B. (**A**) Overlay of monomer A and monomer B of the protease domain (surface of monomer A) in “standard protease orientation” (according to Gomis-Rüth et al., 2012 [38]) (left) and rotated by 90° in “side view” (right). (**B**) Detail view showing the ligand binding within the active site cleft. Residues from monomer A shown in light green and from monomer B in light yellow. The bound zinc ion is shown as a dark grey sphere. The inhibitor MWT-S-270 is shown in stick representation for monomer A, conformation A (dark cyan), monomer A, conformation B (light cyan) and for monomer B (yellow). The zinc ion is penta-coordinated by His^152^, His^156^ and His^162^ and by O1 and O4 of the hydroxamic acid moiety of MWT-S-270 (electron density is shown in Figure S4). One benzoic acid moiety is oriented towards the S1-site, the other towards the S1′-S2′site in both monomers. (**C**) Schematic representation (monomers A, conformation A) of MWT-S-270 bound to the active site of Meprin β. Numbering of inhibitor atoms according to the structure model indicated in blue. The inhibitor consists of a hydroxamic acid linked by a tertiary amine to two symmetric meta-benzoic acid moieties. The hydroxamic acid moiety is bidentately bound to the zinc ion within the active site. One benzoic acid moiety (C7-O16) is oriented toward the substrate-binding site S1 (containing Arg^184^). The other benzoic acid moiety (C17-O26) is oriented towards the substrate-binding sites S1′ (Arg^238^)-S2′ (Arg^146^), whereby hydrogen bonds between the carboxylate of the inhibitor and Ser^212^ occur.

Within the active site in each monomer, a zinc ion is coordinated by the Nε2 atoms of the three histidine residues His^152^ (His^92^ in Astacin), His^156^ (His^96^ in Astacin) and His^162^ (His^102^ in Astacin) (Figure S3E,F). Zinc was identified by strong anomalous scattering at the zinc K_α_ edge (λ_peak_ = 1.2816 Å; measured at beamline i03, Diamond Light Source). In addition to the three histidine residues, the zinc ion is coordinated by the inhibitor MWT-S-270. This compound is composed of a hydroxamic acid moiety linked by a tertiary amine (N6) to two meta-substituted benzoic acid moieties (C7-O16 and C17-O26) (Figure 2C). O1 and O4 of the hydroxamic acid coordinate the zinc as fourth and fifth ligand. The geometry of the coordination is trigonal bipyramidal showing Zn-N coordination distances between 2.09 and 2.21 Å and Zn-O distances between 2.03 and 2.30 Å. Besides the interactions between O1 and O4 of the hydroxamic acid moiety and the zinc ion, a hydrogen bond is observed between N2 of the hydroxamic acid (H-bond donor) and the backbone carbonyl oxygen of Cys^124^. Additionally, an interaction between the hydroxamic acid O1 and the carboxyl side chain of Glu^153^ is present in both monomers (Figure 2 and Figure S4).

The observed electron density supports the presence of the co-crystallized inhibitor MWT-S-270 spanning around the prime site from S1 to the non-prime site region of the proteolytic cleft. However, the density of the inhibitor is different in both monomers, especially for the benzoic acid moieties bound to the tertiary amine (N6) directed either to the S1 site (atom numbering C7-O16) or to the S1′/S2′site (atom numbering C17-O26).

In monomer A, the electron density for the benzoic acid moiety (C7-O16) directed to the S1 subsite is not well defined. Density is observed for C7, C8, C9, C10 of the aromatic ring, but not visible for C11, C12 and C13 of the aromatic ring. From the Omit-map, calculated in the absence of MWT-S-270 (Figure S4), the same result was obtained. Enzyme-inhibitor interactions of this benzoic acid moiety could not be identified within this chain. In monomer B, the same benzoic acid moiety is oriented towards the S1 pocket. Here too, the electron density is not well defined. The inhibitor conformations in chain A and B differ in terms of orientation of the tertiary amine and rotation around the free rotatable C7-C8 single bond. The discussed benzoic acid moiety (C7-O16) is oriented in chains A and B towards the substrate binding pocket S1 of the protease, which is lined by the basic amino acid Arg^184^, albeit the carboxy group of this benzoic acid moiety in chain B is oriented rather towards Cys^124^ than to Arg^184^. The distance between O16 of the benzoic acid moiety of MWT-S-270 in chain A and the guanidino group of Arg^184^ is approximately 4 Å. In chain B, electron density for the sidechain of Arg^184^ is not visible.

A lack of density is also observed for the adjacent residues in S1, Ser^182^-Glu^185^. These residues show higher B factors in chain B than in chain A. Different crystal contacts might provide a rationale for this observation. A possible salt bridge can form between Arg^184^ (chain A) and Asp^368^ (chain B; symmetry operation: -X,Y,-Z). In monomer B, no such interactions could be observed. The lack of clear electron density for the inhibitor and the amino acid side chains in chain B can be interpreted as significant flexibility in this region.

The other benzoic acid moiety of the inhibitor (atom numbering C17-O26) is oriented towards the substrate binding sites S1′ and S2′, which are lined by Arg^238^ and Arg^146^. In chain A, ambiguous density was observed at the aromatic ring of the benzoic acid. The density can be interpreted by two different inhibitor conformations, which differ by rotation around the single bond between C17 and C18. In this model, the inhibitor appears in chain A in two different conformations, A and B. To obtain more information concerning the alternative conformations, omit map calculations in absence of single inhibitor conformers were performed (Figure S5). This analysis confirms the two different conformations of the inhibitor, each being occupied by ~50%. In chain B, only one conformation was built, corresponding to the conformation A in chain A. In this conformation, the inhibitor carboxylate O26 (O25 in chain B) exhibits a hydrogen bond to the peptide backbone (N of Ser^212^) and O25 (O26 in chain B) to the Oγ of Ser^212^ and a water molecule (not incorporated in chain B), which is further linked to Oγ of Thr^214^ (Figure S6). The moiety of the inhibitor is oriented towards Arg^238^ (S1′) with an approximate distance of 4 Å between O26 (O25 in chain B) of the inhibitor and the guanidine group of Arg^238^. In addition to the different orientations of the benzoic acid moieties in monomer A and B, other contacts of the inhibitor appear slightly different. In monomer A, the hydroxylic group of Tyr^211^ from the Met-turn is in proximity (2.9 Å) to C7 of the inhibitor. In Monomer B, the same hydroxylic group is in proximity (3.4 Å) to C17 of the inhibitor, conformation A. Furthermore, the inhibitor density in general is slightly different between both chains. In chain A, the density of the inhibitor seems well defined and supports both inhibitor conformations. In chain B the density seems less well defined, especially surrounding the hydroxamic acid, C5 and the tertiary amine (Figure S4).

**Figure 3:**
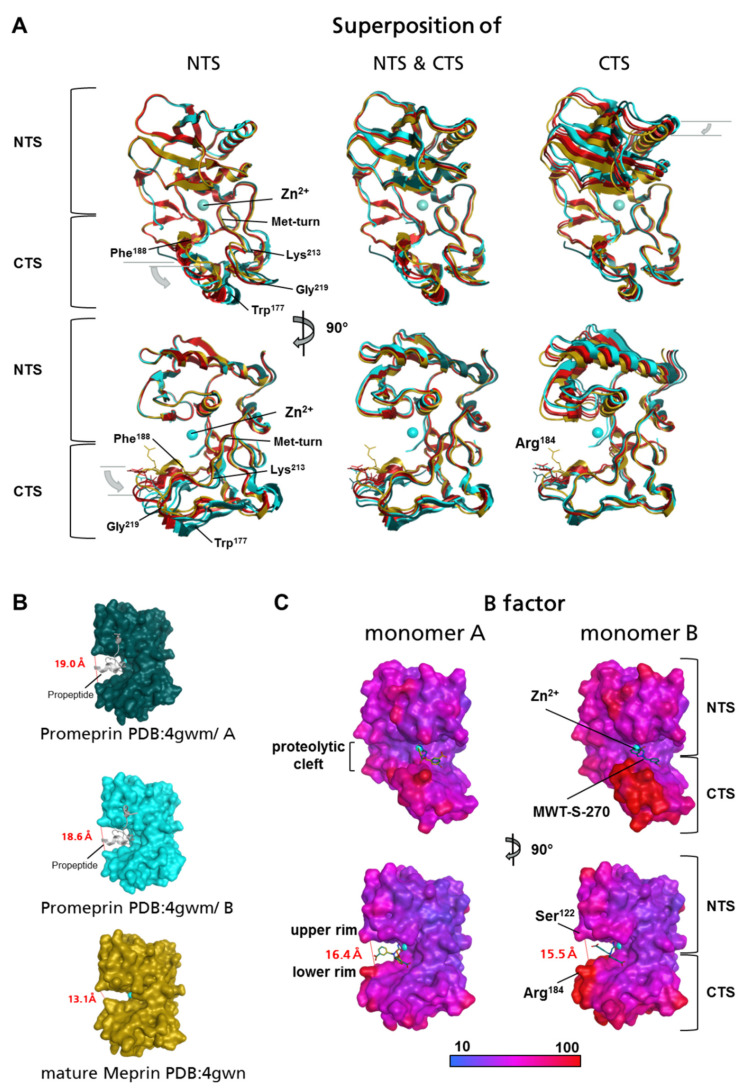
Structural differences of crystallized protease domains of Meprin β. Protease domain of Meprin β in “standard protease orientation” (upper images) and after rotation in the “side view” (lower images). (**A**) Superposition of the protease domains from Promeprin β (PDB:4GWM, pro-peptides are not indicated) monomer A (dark cyan) and monomer B (light cyan), mature Meprin β (PDB:4GWN, gold), MWT-S-270-bound monomer A (light red) and monomer B (dark red). Superposed are N-terminal subdomains (NTS, left), the whole protease domain (NTS & CTS, middle) or the C-terminal subdomains (CTS, right). (**B**) Molecular surface of the protease domains (side view) of Promeprin β (PDB:4GWM) monomer A (dark cyan) and monomer B (light cyan), mature Meprin β (PDB:4GWN, gold). Pro-peptides indicated as ribbon. (**C**) For both monomers (A and B) of inhibitor-bound Meprin β^62–595^ (MβΔC) the molecular surface is shown, colored by B factor.

Due to the presence of alternative binding conformations of MWT-S-270 and a high flexibility at the lower rim of the active site cleft, whether structural changes within the protease domain occur upon pro-peptide-/inhibitor-binding or maturation of the protease (Figure 3) has been investigated. A smaller distance between the upper rim and the lower rim of the active site of mature Meprin β (pdb:4gwn), compared with the pro-form (pdb:4gwm) are in accordance with such an assumption.

The protease domain of Meprin β in complex with MWT-S-270 shows the same secondary structural organization as mature Meprin β (pdb:4gwn), its pro-form (pdb:4gwm) and the prototypic astacin from *Astacus astacus*. The protease domain is divided by the active site cleft into an N-terminal subdomain (NTS) and a C-terminal subdomain (CTS). The N-terminal “upper” subdomain consists of a highly twisted five-stranded β-sheet flanked by two long α-helices on its concave site. The second helix (active site helix; Ile^147^-Leu^158^) contains the N-terminal part of the zinc binding motif (**H**^152^EFL**H**^156^ALGFW**H**^162^E) and ends at the conserved glycin (Gly^159^) with a sharp turn of the polypeptide. This turn allows the chain to enter the “lower” CTS, which is characterized by short 3_10_-helices and 3-turns [39]. The inhibitor-bound Meprin β (monomer A red, monomer B dark red) was compared with both monomer structures of Promeprin β (monomer A dark cyan, monomer B cyan) and the structure of mature unbound Meprin β (PDB:4GWN, gold) (Figure 3A).

Three different superpositions of the protease domains from both inhibitor-bound monomers (A and B), both monomers of the Pro-Meprin β (A and B, blocked by the pro-peptide), and mature Meprin β (native active site without inhibitor) were prepared.

In the first superposition, the whole protease domain (Asn^62^-Leu^259^) was used. In the second and third, only the NST (N-terminal subdomain, Asn^62^-Phe^160^) or the CTS (C-terminal subdomain, Trp^161^-Leu^259^) were considered, respectively. By superposition of the whole protease domain (Asn^62^-Leu^259^) in the NTS (Asn^62^-Phe^160^) as well in the CTS (Trp^161^-Leu^259^) divergences between the different protein variants were observed. In the second superposition of the NTS, the first five amino acids of the protease domain were excluded: only Glu^67^-Phe^160^ were used for the superposition and the relative orientation of the NTS to CTS was kept. This analysis did not reveal any differences (RMSD 0.25 Å) in the NTS. In contrast, positional flexibility was observed within the CTS, as supported by the RMSD of 1.95 Å. A minor change was observed in this analysis concerning the three-turn spanning Lys^213^-Gly^219^, C-terminal of Met-turn (S^207^-Y^211^). In the middle part, an RMSD (Cα atoms) of 1.9 Å was observed between all five investigated structures. A more drastic change was seen in the region spanning two short CTS-characteristic 3_10_-helices, Trp^177^-Arg^179^ and Glu^185^-Phe^188^, and especially the intermediate three-turn spanning Leu^181^-Gly^183^. In this three-turn, the positions differ clearly as suggested by an overall RMSD (Cα atoms) of 3.8 Å. This part displays the most significant differences between the monomers in the structure presented here and those published previously. In Promeprin β, Arg^184^ interacts with Glu^42^ of the pro-peptide, but in the inhibitor-bound Meprin β as well as mature unbound Meprin β no substituting interaction could be observed. In the inhibitor-bound Meprin β, Trp^177^-Phe^188^ seems to be shifted, as seen for Lys^213^-Gly^219^, towards the position found in Promeprin β. The strongly shifted Arg^184^, which juts directly into the active site cleft in mature unbound Meprin β (shown as golden sticks), is shifted more than 5 Å out of the active site. This leads to an increased opening of the cleft containing the active site and of the corresponding position in the inhibitor- and in the pro-peptide-bound form. By superposition of the CTS, the NTS is shifted with an RMSD of 1.96 Å, but again differences in the three-turn from the CTS, harboring Arg^184^, were observed. In summary, the superpositions led to the conclusion that both subdomains, NTS and CTS, have a distinctly different relative orientation to each other dependent on the binding state (non-/inhibitor-/pro-peptide-bound). Thus, the connection between both subdomains (Phe^160^-Trp^161^) appears to act as a hinge region (hinge motion). High flexibility (B-Factors) was observed especially for the three-turn, spanning Lys^213^-Gly^219^, and Trp^177^-Phe^188^ close to the lower rim of the catalytic cleft (Figure 3C).

### 2.3. Interaction Analysis of Meprin β^62–595^ (MβΔC) and MWT-S-270 by Calorimetry and Molecular Dynamics (MD)–Simulations

Previous studies of the SAR of hydroxamate-based inhibitors aimed at targeting of arginine residues present in the active site cleft of Meprin β. Indeed, the carboxy-substituted benzoic rings in MWT-S-270 mediate a more than 100-fold stronger inhibition in comparison to Meprin α [33]. Because the results of the complex structure could not be interpreted in terms of presence of multiple salt bridges, we employed ITC to study the dependence of the dissociation constants on the ionic strength of the solvent and, thus, if any electrostatic contributions can be observed with regard to inhibitory binding. In particular, we intended to study a contribution of the benzoic acid moiety (atoms C7-O16) to the binding at the S1 subsite (Arg^184^). Differently substituted inhibitors were selected for the interaction analysis (Table 2, Figure S7). All inhibitors exhibit the hydroxamic acid group bound by the Meprin β monomers, the tertiary amine, as well one benzoic acid moiety. The other benzoic acid of MWT-S-270 was exchanged by a non-charged dioxolane moiety (2) or a non-polar, non-charged phenyl moiety (3). Legible from the ITC binding curves, in all assessments inhibitor binding occurred with one inhibitor molecule bound to one protein molecule. Binding affinities drop from 16 nM (ΔH = −3884 cal/mol; −ΔS * T = −6939 cal/mol) for MWT-S-270 to 237 nM (ΔH = −3966 cal/mol; −ΔS * T = −5212 cal/mol) for the non-charged dioxolane substituted variant and further to 746 nM (ΔH = −1138 cal/mol; −ΔS * T = −7363 cal/mol) for the non-polar, non-charged phenyl substituted variant. An increase of the salt concentration from 0 mM to 150 mM increased the dissociation constant from 16 nM to 400 nM (ΔH = −4554 cal/mol; −ΔS * T = −4303 cal/mol) supporting electrostatic interaction(s) as a contributor to binding of MWT-S-270 [40].

**Figure 4:**
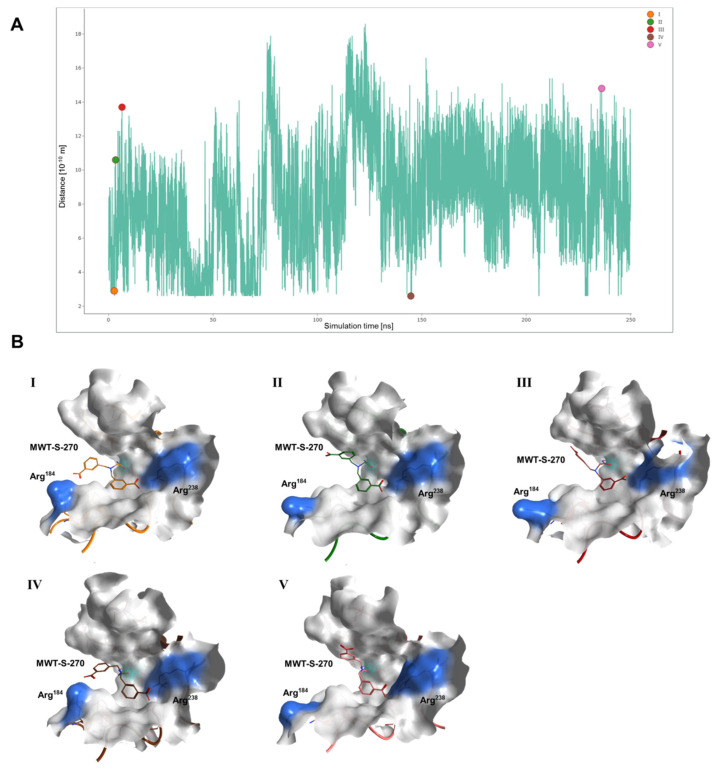
Molecular dynamic (MD) simulations to analyze possible interaction patterns between the inhibitor MWT-S-270 and Meprin β^62–595^ (MβΔC). (**A**) Distance Plot between guanidine Ns of Arg^184^ and O15/O16 of MWT-S-270 over the 250-ns MD. (**B**) Five conformations, which were extracted from the MD simulation (refer to the colored markers in (**A**)).

In order to study potential interaction patterns between the inhibitor MWT-S-270 and Meprin β^62–595^ (MβΔC) in more detail, we employed molecular dynamics simulations. The main focus was set to the fluctuating distances between the benzoic acids of MWT-S-270 and the corresponding binding partners forming the S1 and S1′ sub-pockets. Therefore multiple, independent simulations (identical parameter) were performed and the resulting trajectories with mutable simulation times (between 120 nano seconds [ns] and 500 ns) were analyzed. From these and other calculations (not shown) we conclude a high flexibility of the residues, shaping the lower rim (CTS) of the S1 pocket (with Arg^184^ as central residue), numerically calculated by RMSF-values between 1.4 Å and 2.27 Å (Figure S8). The frame-wise calculation of distances between the carboxylic oxygens (O15 & O16) from MWT-S-270 and the guanidine nitrogens from Arg^184^ show, that beside the fluctuation of the Arg^184^ sidechain, an additional rotation of the benzoic acid (moiety C7-O16) occurs, providing the cause for the increasing and decreasing distances over time (Figure 4A).

**Figure 5:**
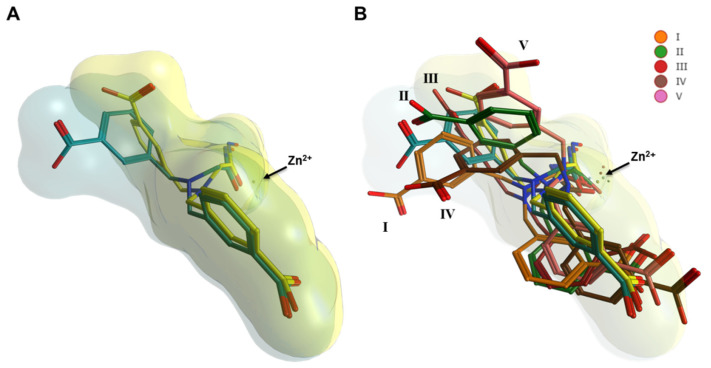
Overlay of experimental estimated and MD simulated MWT-S-270 within the active site of Meprin β^62–595^ (MβΔC). (**A**) Overlay of co-crystallized ligand MWT-S-270 for monomer A, conformation A (dark cyan) and for monomer B (yellow). Both ligands are depicted as sticks, surrounded by its specific molecular surface, colored according to stick-color. (**B**) MD-snapshot-ligands I–V from Figure 4B overlayed with (**A**).

In addition, five snapshots were extracted from the trajectory (marked as colored circles I-V) and separately visualized in Figure 4B, and also as overlay in Figure 5. The direct comparison of the co-crystallized inhibitor with MD-snapshots poses structure II as most similar to the Inhibitor (C7-O16, both conformers), resolved in monomer A (pairwise RMSD of 1.7 Å). Structure V from Figure 4B is very close to the conformation of the inhibitor in monomer B (pairwise RMSD of 1.9 Å). An analysis of the distances of the second benzoic acid (atoms C17-O26) which faces into the S1′ pocket clearly shows a very stable orientation, mainly to its counterpart Arg^238^ and the surrounding H-Bond network, after 90 ns (Figure S9). The metal coordination of the hydroxamic acid remains very stable in all simulations over time. Hence, the MD simulations provide a rationale for the conformations of MWT-S-270 observed in the crystal structures.

## 3. Discussion

A characteristic feature of astacin metalloproteinases is their remarkable preference for acidic amino acids in P1′-positon of the substrates [41]. The structural basis for this preference is a highly conserved arginine residue, shaping the bottom of the S1′-subsite of the proteinases [23]. In particular, Meprin β exhibits a unique substrate specificity to cleave peptide substrates only composed of acidic amino acids. These cleavage characteristics are mediated by a cluster of positively charged residues within the active site cleft [21,41]. In addition to the conserved arginine Arg^238^ in the S1′-pocket, the S1- as well as the S2′-subsites of Meprin β are formed by the arginines Arg^184^ and Arg^146^, respectively.

More recently, the first synthetic small molecule inhibitors of Meprin α and β have been developed and their structure–activity relationships (SAR) have been elucidated in detail [31,32,33]. These studies suggested that acidic moieties are mandatory for high inhibitory potency against Meprin β and are also a key structural feature for high selectivity over Meprin α or other related proteinases, e.g., MMPs and ADAMs. To obtain more detailed insight into the binding mode of these inhibitors, we co-crystallized Meprin β^62–595^ (MβΔC) with the selective and potent Meprin β inhibitor MWT-S-270 and solved the structure of the complex by X-ray crystallography. The obtained structure revealed binding of the inhibitor in both monomers within the active site cleft (Figure 2). As predicted by the docking studies [31], MWT-S-270 is a “right-hand side” inhibitor addressing the S1 and S1′-sites of Meprin β, thus behaving like the majority of hydroxamate-based metalloproteinase inhibitors. The hydroxamic acid moiety is bound to the zinc ion in a bidentate fashion, which is commonly observed with these inhibitors. One benzyl residue is directed to the S1-site, while the second benzyl residue of the tertiary amine is directed towards the S1′-subsite, shaped by Arg^238^. The electron density of this residue is defined in both monomers of the dimer, suggesting interactions of the benzyl moieties within this sub-pocket. The postulated interaction of the carboxylic acid with Arg^238^ does not seem to generate the main commitment to binding of the inhibitor at the S1′-site, exhibiting a closest distance of 3.9 Å between the oxygen of the carboxylic acid and N of the guanidinium moiety of the arginine side chain (Figure S4). In addition, in monomer A of Meprin β, we observed a second conformation of MWT-S-270 (conformer B), which is located at a distance of 6.7 Å to the S2′ side residue Arg^146^ (considering the oxygen of the carboxylic acid and N of the guanidinium moiety of the arginine-sidechain).

The unexpected interaction is a fixation of the benzoic acid moiety within the S1′-site via a network of hydrogen bonds, i.e., the sidechain OH of Ser^212^ at a distance of 2.8–2.9 Å, the backbone NH of Ser^212^ at a distance of 3.2 Å and a water-bridged hydrogen bond with Thr^214^ at a distance of 2.6 and 2.8 Å. However, this unexpected hydrogen bond network might not only be a contributor to the affinity of the inhibitor, but also to its selectivity over Meprin α. The respective residues in Meprin α are Gln^215^ instead of Ser^212^ (Meprin β) and Phe^217^ instead of Thr^214^ (Meprin β) (Figure S10). Thus, the two hydrogen bond donors are replaced by bulkier residues, lacking hydrogen bond donors. This narrows the S1′-pocket sterically and hampers formation of the hydrogen bond network, finally leading to decreased inhibitory potency of MWT-S-270 towards Meprin α (IC_50_ = 16,050 ± 212 nM) [31].

While the same interaction of MWT-S-270 with the S1′-subsite occurs in both monomers of the dimer, the position of the benzyl residue that interacts with the S1-subsite is less defined. The closest distance between the carboxylic acid and the guanidinium moiety of Arg^184^ is around 4 Å, albeit the electron density for the C11, C12 and C13 of MWT-S-270 in monomer A and C8, C9 and C13 in monomer B is not defined, and the moiety of the inhibitor is probably highly flexible via rotation around the single bond connecting C7-C8. Moreover, the whole loop forming the lower rim of the active site cleft, harboring Arg^184^, is also less well resolved, as reflected by higher B-factors, especially in monomer B. The missing electron density of the side chain of Arg^184^ in monomer B and the barely resolved Arg^184^ in monomer A (together with the less well resolved whole loop) led us to conclude a high degree of flexibility. Because the crystal structure data do not allow a reliable evaluation of MWT-S-270 interactions with the S1-pocket, we performed molecular dynamics (MD) simulations. In these simulations, we see the high flexibility of the residues, shaping the lower rim (CTS) of the S1 pocket and an additional rotation of the benzoic acid of the inhibitor, with increasing and decreasing distances over time. Especially the high flexibility of the solvent-exposed residue Arg^184^, but also the proposed intermediate conformations of MWT-S-270, which “fills the gap” between the two states in the crystal structure model (monomer A and B), could be illustrated by MD (Figure 4). An interesting result is the direct comparison of MD–snapshot II with the inhibitor interpreted in monomer A from the X-ray structure, where the S1-subsite directed benzyl residues are close to each other but the Arg^184^ sidechain moves out of the cleavage site, allegedly with high solvent accessibility and associated polarization effects. The MD–snapshot IV contrasts, in that here the benzoic acid moiety is found in similar orientation as in snapshot II, but Arg^184^ faces the inhibitor directly with remarkably reduced distance, in comparison also with the crystal structure. Taken together, conformations II and IV support the theory of high flexibility and, therefore, weakly defined electron densities. In particular Arg^184^, one of the putative key residues for electrostatic interaction, exhibits high flexibility, which is even increased in the presence of the inhibitor. Thus, the flexibility of the protein in combination with the flexibility of the inhibitor might be the reason for the weakly defined electron density of the S1-site.

Nevertheless, electrostatic interactions appear crucial for the high affinity of the Meprin β inhibitors and a directed interaction. Such a conclusion is supported by the comparison of the thermodynamics of binding of differently substituted inhibitors and the influence of ionic strength on the inhibition. Assuming a conserved interaction of the benzoic acid with the S1′-subsite, the three analyzed compounds (Table 2) differ in the residue that potentially addresses the S1-pocket. While the carboxylic acid of **1** could participate in ionic interactions and charged hydrogen bonds, the dioxolane moiety of **2** may just serve as hydrogen bond acceptor and the unsubstituted phenyl-residue of **3** does not support an electrostatic interaction with S1, e.g., Arg^184^. The K_i_ values of compounds **1**–**3** support the formation of a directed interaction to S1 (Table 2), as concluded from the drop of the inhibition constant (K_i_) of 23 nM for **1** to 1395 nM for **3**. Thereby, the interaction of **1** is significantly driven by electrostatic interactions, which could be shown by a strong decrease of affinity in presence of 150 mM NaCl (16 nM vs. 400 nM), at which the lower affinity was not caused by a weaker binding enthalpy (ΔH) (−3884 cal/mol vs. −4554 cal/mol), but the loss of favorable binding entropy (−ΔS * T) during the interaction (−6939 cal/mol vs. −4303 cal/mol). In this regard, it has to be considered that the contribution of ionic forces to the binding is dependent on the environment. In an a-polar environment (e.g., interior of proteins) the formation of salt bridges is mainly driven by enthalpic contributions opposed to entropic penalties, whereas in a polar environment (e.g., surface of the protein) the ionic interactions are mainly driven by an entropic gain [42,43,44]. Accordingly, the favorable entropic contribution of **2** is significantly lower compared to **1**. Further decreased affinity of **3** is mainly caused by the weakest binding enthalpy of all three compounds and only slightly compensated by a small entropic gain, which indicates that removal of the functional group caused the loss of directed interaction. Although the interaction partner within the S1-subsite could not be unambiguously identified by our studies due to the high flexibility, the data obtained here support the concept that interactions within the S1-subsite, e.g., Arg^184^ in Meprin β, Tyr^187^ in Meprin α, Phe^214^ in Ovastacin and Gln^246^ in BMP-1, could be a key to the development of selective inhibitors for the respective proteinases.

In addition to the S1 pocket including the sidechain of Arg^184^ and its surrounding residues, the whole lower rim of the CTS also shows a remarkable flexibility, which is supported by high B-factors for this C-terminal subdomain, especially in Monomer B (Figure 3C). Compared with the previously reported mature Holo-Meprin β (PDB:4GWN; [21]), the active site cleft of the inhibitor-bound Meprin β is distinctly broadened. The distance between side chain Oγ of Ser^122^ (outer end of the upper rim) and the guanidinium Nε of Arg^184^ (outer end of the lower rim) in the inhibitor-bound Meprin β is ~11 Å (monomer A; not determined for monomer B due to lack of density for the Arg^184^ side chain) but in the mature Holo-Meprin β this is only ~8 Å. Distances of ~15 Å (monomer A and B) are observed in Promeprin β (PDB:4GWM). Such differences, although less pronounced, are also recognized for the Cα trace. For instance, the distances of Cα of Ser^122^-Cα of Arg^184^ for inhibitor-bound Meprin β monomer A were 16.6 Å, in monomer B 15,7 Å, in Holo-Meprin β 13.1 Å, in Promeprin β (PDB:4GWM) Monomer A 19 Å and in Monomer B 18.6 Å (Figure 3B,C). Such alterations of the positions between NTS and CTS are observed in all Astacins, which have been analyzed so far. Only in human bone morphogenetic protein 1 (BMP1) is the active site cleft in the inhibitor-bound form (PDB:6BTN) more constricted in comparison with the non-inhibitor bound form (PDB:3EDG). Nevertheless, a movement/changed position is also observed there.

The superposition of Promeprin β, mature Meprin β and MWT-S-270-bound Meprin β protease domains revealed a switch in the relative orientation of the subdomains to each other, whereby the connection (Phe^160^-Trp^161^) between both subdomains may act as a hinge. For the single domain astacin from *Astacus astacus*, a slight hinge movement (~1 Å) upon inhibitor binding has also been reported [27]. In Meprin β, the respective region (Lys^213^-Gly^219^ and Trp^177^-Phe^188^) within the CTS did not develop additional protein–protein interactions upon structural change and the CTS remains solvent accessible. This contrasts with the NTS, which displays pronounced interactions with the MAM and TRAF domain as well as with the respective other monomer of the dimer. Hence, it is tempting to speculate that the CTS is the moving subdomain during binding events.

Concluding, we propose the following structural changes occurring in Meprin β upon activation and ligand/substrate binding. Promeprin β harbors a broader cleft of 15 Å between the upper (NTS) and the lower rim (CTS) due to pro-peptide binding. Caused by pro-peptide cleavage and release, the distance between the upper and the lower rim is reduced by ~7 Å. The newly formed active site cleft remains flexible, and may be broadened again upon inhibitor binding. Thereby, the size of the cleft depends on the structural orientation of the bound ligand, in our example this is influenced by the free rotation around the single bond connecting C7 and C8 of MWT-S-270. Depending on the type of ligand, the active site cleft maybe adjusted by hinge movement between NTS and CTS, thereby exerting an induced fit mechanism. Such a theory could be further corroborated by crystallization in complex with a more rigid ligand to fix a preferred conformation.

## 4. Materials and Methods

### 4.1. Expression and Purification of Mature Meprin β^62–595^ (MβΔC)

Active Meprin β^62–595^ (MβΔC) was produced, similar to that described by Schlenzig et al. [34]. The sequence for the C-terminally truncated pro-Meprin β (pMβΔC), spanning the amino acids 23–595 (pro-peptide-, catalytical, MAM- and TRAF-domain) was cloned into vector pPICZαC (Cla1/Not1) downstream to sequences encoding an N-terminal His-tag and the α-mating factor from Saccharomyces cerevisiae. The linearized expression vector was used for transformation of *Pichia pastoris* X33 cells. After clonal selection, cells were grown in a 5 L bioreactor (Biostat B, Sartorius BBI Systems GmbH, Göttingen, Germany), according to the Pichia Fermentation Process Guidelines (Life Technologies GmbH, Darmstadt, Germany). The heterologous expression was driven by the AOX-promoter, enabling the expression of N-His-Meprin β^23–595^ upon feeding of methanol. The fermentation process was stopped after 72 h and the supernatant harvested. The recombinant Meprin was captured by chromatography on a Ni-chelating resin applying expanded bed adsorption. This was followed by cleavage of the His-tag and the pro-peptide by trypsin (bovine Trypsin, Sigma Aldrich, St. Louis, MO, USA). For removal of trypsin and denatured protein from the cleavage product (Meprin β^62–595^), hydrophobic interaction chromatography (Butyl-Sepharose column, 25 × 100 mm; GE Healthcare, Chicago, IL, USA) was used. Finally, mature Meprin β^62–595^ was purified by size exclusion chromatography (Superdex 75 column, 26 × 850 mm; GE Healthcare) and eluted in 30 mM Tris pH 7.6, containing 100 mM NaCl. Typically, 7 mg of homogenous mature Meprin β^62–595^ could be isolated from one liter of fermentation supernatant.

### 4.2. Deglycosylation of Meprin β^62–595^ (MβΔC)

For the removal of surface-exposed glycosyl chains, a deglycosylation was performed prior to crystallization. Typically, purified Meprin β^62–595^ (MβΔC) has been deglycosylated by Endo H (500 U/mg Meprin β) at a concentration of 0.5 mg/mL at 37 °C under non-denaturing conditions (50 mM sodium acetate, pH 6.0) for 4 h. Afterwards, the deglycosylated Meprin β^62–595^ (MβΔC) was concentrated to 19.75 mg/mL applying VivaSpin^®^6 (Sartorius, cut off 10 kDa) centrifugal concentrators. Finally, the proteolytic stability of the concentrated Meprin β^62–595^ (MβΔC) was tested for up to 15 days at 15 °C in a 30 mM Tris pH 7.6, 100 mM NaCl-buffer with and without inhibitor (unspecific inhibitor: actinonin; specific: MWT-S-270) and the sample analyzed by SDS-PAGE.

### 4.3. Determination of Meprin β Activity

The enzymatic activity of Meprin β was determined, essentially as described previously [34,45]. The assay is based on the change of fluorescence intensity due to cleavage of the internally quenched peptide substrate Abz-YVAEAPK(Dnp)G-OH (λ_ex_ 340 nm/λ_em_ 420 nm). For activity determination during expression and purification, the proenzyme was activated by trypsin. For activation, 100 µL of cleavage buffer (50 mM Tris/HCl, 10 mM CaCl_2_, pH 7.5), 50 µL Meprin β solution and 50 µL bovine trypsin 500 µg/mL (Sigma Aldrich) were mixed and incubated for 15 min at 30 °C. An aliquot of the reaction (50 µL) was mixed again with 150 µL assay buffer consisting of 40 mM Tris/HCl, pH 8.0, 0.05% Brij 30. The reaction was initiated by addition of 50 µL of substrate (final concentration 50 µM), which was dissolved in assay buffer.

### 4.4. Crystallization of Meprin β^62–595^ (MβΔC), Data Collection and Structure Elucidation

The inhibitor MWT-S-270, which was applied for co-crystallization, was synthesized as described by Ramsbeck et al. [31]. For crystallization trials, the purified and deglycosylated Meprin β^62–595^ (MβΔC) was combined with the inhibitor MWT-S-270 at a molar ratio of 1:1.2 and a final protein concentration of 8 mg/mL in 30 mM Tris, 100 mM sodium chloride, pH 7.6. Crystals were grown at 13 °C using the sitting drop vapor diffusion technique by mixing 200 nL of protein solution with 200 nL of reservoir buffer. This solution was equilibrated against 55 µL of reservoir buffer containing 25% (*w*/*v*) PEG 4000 and 30% (*v*/*v*) ethylene glycol in the reservoir. Crystals were harvested after 39 days. Prior to X-ray analysis, crystals were flash frozen at −180 °C in an X-Stream 2000 cryo stream (Rigaku/MSC) without further cryoprotection. Diffraction images of a Meprin β single crystal were collected in-house using Cu K_α_ radiation (λ = 1.5418 Å) provided by a copper rotating-anode source (RA Micromax 007, Rigaku Europe) using a CCD detector (SATURN 944+, Rigaku Europe). Oscillation images were integrated, merged, and scaled using XDS to a resolution of 2.41 Å [46] according to an I/Sigma(I) of 1.8 in the highest resolution shell (Table 1). The phases were determined by molecular replacement with the program PHASER [47] using the protein data bank (PDB; https://www.wwpdb.org/ (accessed on 5 May 2021)) entry 4GWN (human mature Meprin β, hexagonal crystal form) as search model. The monoclinic crystal contained two monomers in the asymmetric unit (space group C2). Initial automated model building and refinement were performed using the program AUTOBUILD from the PHENIX suite [48]. Further iterative cycles of manual model building and maximum-likelihood structure refinement were conducted using the programs COOT from the CCP4 suite [49] and PHENIX.REFINE including Non-Crystallographic Symmetry (NCS) and finally also Translation Libration Screw-motion (TLS) restraints. Non-standard ligand restraints for the inhibitor MWT-S-270 were generated with ELBOW (PHENIX suite). Bond lengths of MWT-S-270 were additionally restrained using data contained in the Cambridge Structural Database (CSD). The final model comprises the residues 62–594 for both monomers. In both chains, the inhibitor was built, based on the observed electron density. In protein monomer A, an ambiguous electron density could be a result of two alternative conformations, consequently both conformations were built and refined against occupancies. Remaining oligosaccharide residues were built manually into the electron density in accordance with the known glycosylation pattern of the expression host system *Pichia pastoris* [50,51]. The whole protein model was validated by MolProbity included in the PHENIX suite. Metal ions were validated using the dedicated Metal Binding Site Validation Server (CMM; http://csgid.org/csgid/metal_sites (accessed on 5 May 2021)) [52]. Finally, glycosyl chains were checked using the program Privateer [49,53] and interfaces were analyzed using PISA [54] of the CCP4 suite. Structure coordinates und reflection data were deposited in the PDB under accession code 7AQ1. The inhibitor MWT-S-270 was also deposited as chemical compound in the PBD under 3-letter-code RUE (CC ID). Figures were prepared using Molecular Operating Environment (MOE) v.2019.0102 (Chemical Computing Group ULC).

### 4.5. Isothermal Titration Calorimetry (ITC)

The binding affinity (K_D_), binding enthalpy (ΔH), binding entropy (ΔS) and stoichiometry (N) of different Meprin β inhibitors to Meprin β^62–595^ (MβΔC) were determined by isothermal titration of the inhibitors to the enzyme at 30 °C using a VP-ITC microcalorimeter (MicroCal). Prior to the binding analysis, the purified Meprin β^62–595^ (MβΔC) was dialyzed extensively against 40 mM Tris pH 8.0 (±150 mM NaCl). The inhibitors were diluted 1:50 from DMSO stock solutions into dialysis buffer and 2% (*v*/*v*) DMSO were added to the dialyzed enzyme solution immediately before starting the titration experiment. The instrument was operated according to the manufacturer’s instructions and the data analyzed using MicroCal ORIGIN software. The obtained binding heat was corrected by the dilution heat of the ligand, which was recorded by titration of the inhibitor (1:50 diluted) into the dialysis buffer containing 2% (*v*/*v*) DMSO. The corrected data were evaluated by a single-site binding model calculating N, K_D_, ΔH and ΔS.

### 4.6. Molecular Dynamic (MD) Simulations

Monomer A of PDB entry 7AQ1 was used for MD simulations. All sugar and water molecules were removed and only protein residues, ions (including active site Zn^2+^) and the ligand MWT-S-270 were kept. The system was protonated at pH 7.0 using Protonate 3D [55]. The parameter and library files of the small molecule ligand were generated by the antechamber and parmchk tools, using the Am1-BCC charge model for atomic point charges in combination with gaff atom types. The amber14 force field [56] was employed for the protein and ions. The ligand, protein and ions were merged into a new file and the resulting complex was inserted into a TIP4PEW [57] water box. Furthermore, the system was neutralized by adding counter ions and disulfide bridges were defined manually. It is well known that the non-bonded simulation of explicit water and ligand molecules, bound to catalytic metal ions, is challenging due to the underestimation of the interactions between water and metal [58]. Therefore, some adoptions were performed to utilize an extended lennard-jones-C4 Potential [59] as non-bonded model with fine-tuned m12-6-4 parameter set [60]. All calculations were executed on two Nvidia GeForce RTX 2080 Ti GPUs, which are part of an Intel Core I9 (14 × 3.3 GHz) high performance workstation (HPW) with 128 GB RAM, running Ubuntu 18.04. All trajectories were processed using cpptraj [61] and subsequently analyzed with VMD [62,63,64] and RStudio-Server (V1.3.959 [65]; R V3.6.3 [66]), utilizing Bio3D [67,68,69] and Plotly [70].

## 5. Conclusions

In this work we present the first structure of Meprin β in complex with a small molecule inhibitor. The data support an unexpected flexibility of the compound MWT-S-270 within the active site and provide evidence for a general flexibility of the active site region. The results will have implications for the development of novel Meprin inhibitors, as already demonstrated [71]. However, the high flexibility of MWT-S-270 as well as the active site needs to be considered in future structure based drug design approaches.

## Figures and Tables

**Table 1:** Collection and refinement statistics. (Statistics for the highest-resolution shell are shown in parentheses).

	Meprin β/MWT-S-270
**Data collection statistics**	
Radiation source	Rotating anode
Wavelength (Å)	1.5418
Space group	C 1 2 1
Unit cell length (Å)	162.25, 72.44, 135.47
Unit cell angles (°)	90, 118.43, 90
Resolution range (Å) Highest resolution shell (Å)	50–2.41 2.48–2.41
R_meas_	13.1 (112.9)
I/σI	11.48 (1.79)
Completeness (%)	99.1 (95.5)
CC (1/2) Multiplicity	99.7 (74.6) 5.6 (5.1)
Solvent content/Meprin β per ASU	58%/2
Wilson B factor	41.01
**Refinement statistics**	
Number of reflections (working/test set)	103,499/ 5195
R_work_/R_free_	0.20/0.24
No. atoms	
Protein	8542
Ligand	431
Water	417
Average B-factors (Å^2^)	52.27
Protein	51.77
Ligand	68.94
Water	45.31
Bond length r.m.s.d. (Å)	0.003
Bond Angles r.m.s.d. (°)	0.63
Ramachandran plot (%): favored/allowed/outliers	97.8/1.98/0.19
MolProbity clashscore	0.75

R_meas_ = redundancy independent indicator of data quality [36].

**Table 2:** Enzyme inhibition and thermodynamic profiling of Meprin β inhibitors. (ITC binding curves for the titration of Meprin β^62–595^ (MβΔC) with the inhibitors is given in supplementary Figure S7).

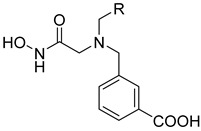		ITC Buffer	IC_50_ [nM]	K_i_ [nM]	K_D_ [nM]	ΔH [cal/mol]	−ΔS * T [cal/mol]
	**R**						
**1** (270)	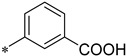	40 mM Tris pH 8.0	45,5	28,6	16	−3884	−6939
**1** (270)	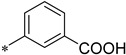	150 mM NaCl, 40 mM Tris pH 8.0	176	198	400	−4554	−4303
**2** (416)	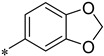	40 mM Tris pH 8.0	285	192	237	−3966	−5212
**3** (396)	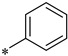	40 mM Tris pH 8.0	1283	1395	746	−1138	−7363

## Data Availability

Structure coordinates und reflection data of Meprin β^62–595^ (MβΔC) homodimer (Meprin B) crystal structure in complex with MWT-S-270 has been deposited at PDB (protein data bank; https://www.wwpdb.org/ (accessed on 5 May 2021)) under accession code 7AQ1. The inhibitor MWT-S-270 has been deposited as chemical compound as RUE (CC ID).

## Supplementary Materials

The following are available online at https://www.mdpi.com/article/10.3390/ijms22115651/s1.
