# Supplementary material for: Structure and Dynamics of Meprin β in Complex with a Hydroxamate-Based Inhibitor
Source: Int J Mol Sci. 2021 May 26;22(11):5651. doi: 10.3390/ijms22115651 (PMC8197800; doi:10.3390/ijms22115651)
Supplement: Supplementary file 1 [file ijms-22-05651-s001.zip › ijms-1230236-supplementary.pdf]

Supplemantary Information for

Structure and Dynamics of Meprin β in Complex with a Hydroxamate-based Inhibitor

Miriam Linnert, Claudia Fritz, Christian Jäger, Dagmar Schlenzig, Daniel Ramsbeck, Martin Kleinschmidt, Michael Wermann, Hans-Ulrich Demuth, Christoph Parthier, Stephan Schilling

This PDF file includes:

Figure S1: SDS-PAGE analysis of Meprin β expression and purification.

Figure S2: SDS-PAGE analysis of purified, activated Meprin $\beta^{62\text{-}595}$ (MβΔC).

Figure S3: Metal binding sites in Meprin B structure.

Figure S4: MWT-S-270 bound to the active site of Meprin β.

Figure S5: Omit electron density map in the absence of MWT-S-270 in chain A.

Figure S6: S1´-subsite interactions of Meprin β with MWT-S-270 (shown for monomer A, conformation A).

Figure S7: ITC binding curves for the titration of Meprin $\beta^{62\text{-}595}$ (MβΔC) with different inhibitors.

Figure S8: The RMSF of Cα atoms of Meprin $\beta^{62\text{-}595}$, calculated from a 250-ns MD simulation.

Figure S9: Distance between the guanidine nitrogens of Arg^{238} and the interaction partner (O25 & O26) of MWT-S-270 over 250 ns MD.

Figure S10: Comparison of the structure of Meprin β with an homology model of Meprin α.

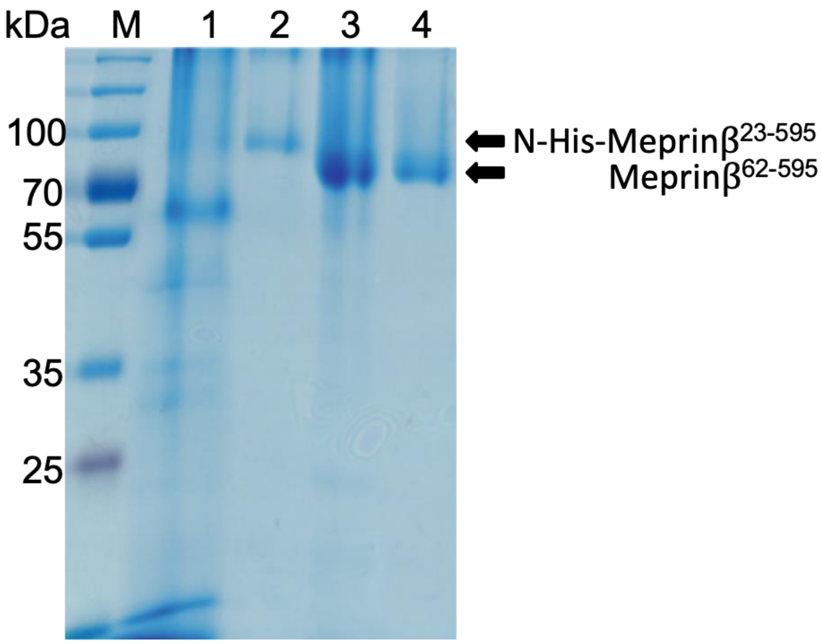

Figure S1: SDS-PAGE analysis of Meprin β expression and purification. Samples were separated using a 12 % Tris-glycine gel and visualized by Comassie staining. (1) Supernatant of fermentation broth, (2) after chromatography on immobilized metal ions (IMAC), (3) after hydrophobic interaction chromatography (HIC), (4) after size exclusion chromatography (SEC). Following initial purification by Ni-IMAC, Meprin β was activated using trypsin, leading to a mass shift due to removal of the propeptide (compare to Figure 1A).

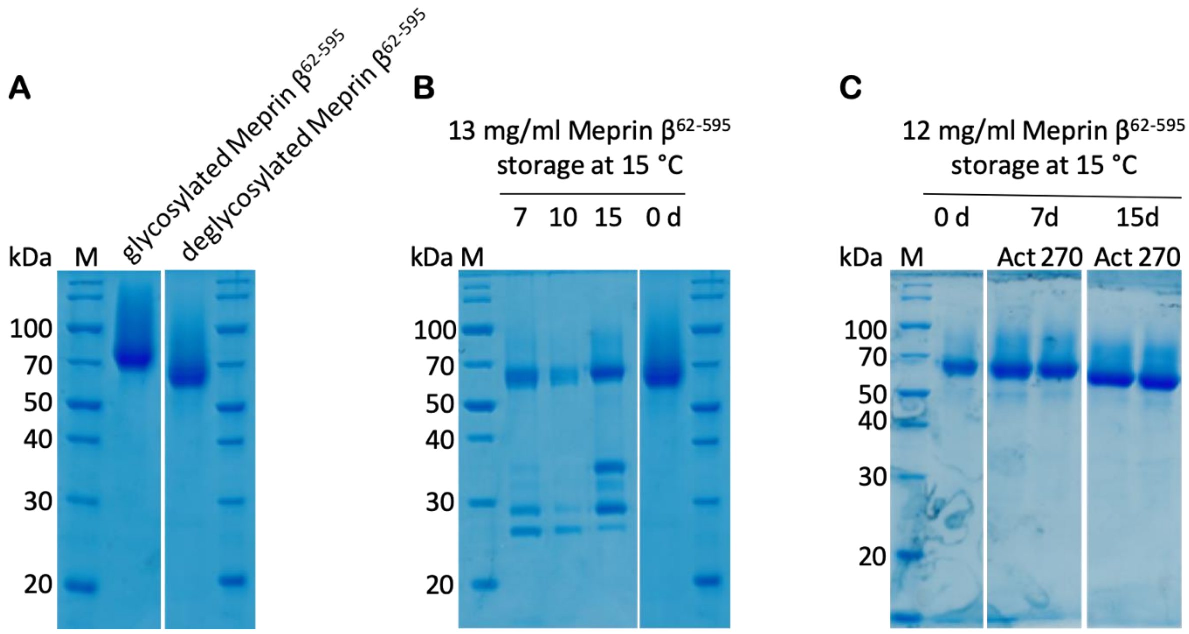

Figure S2: SDS-PAGE analysis of purified, activated Meprin $\beta^{62\text{-}595}$ (MβΔC). A) Meprin $\beta^{62\text{-}595}$ (MβΔC) before and after deglycosylation by EndoH under native conditions. The mass shift indicates a successful partial deglycosylation. **B)** Proteolytical stability of highly concentrated Meprin $\beta^{62\text{-}595}$ (MβΔC) for up to 15 days at 15°C. Degradation of Meprin β was observed after 7 days, possibly due to autocatalytic activity [35]. **C)** To increase proteolytic stability of mature, deglycosylated Meprin $\beta^{62\text{-}595}$ (MβΔC), the commercial inhibitor actinonin (Act) and the specific inhibitor MWT-S-270 (270) have been added, leading to stability at 15°C for at least 15 days.

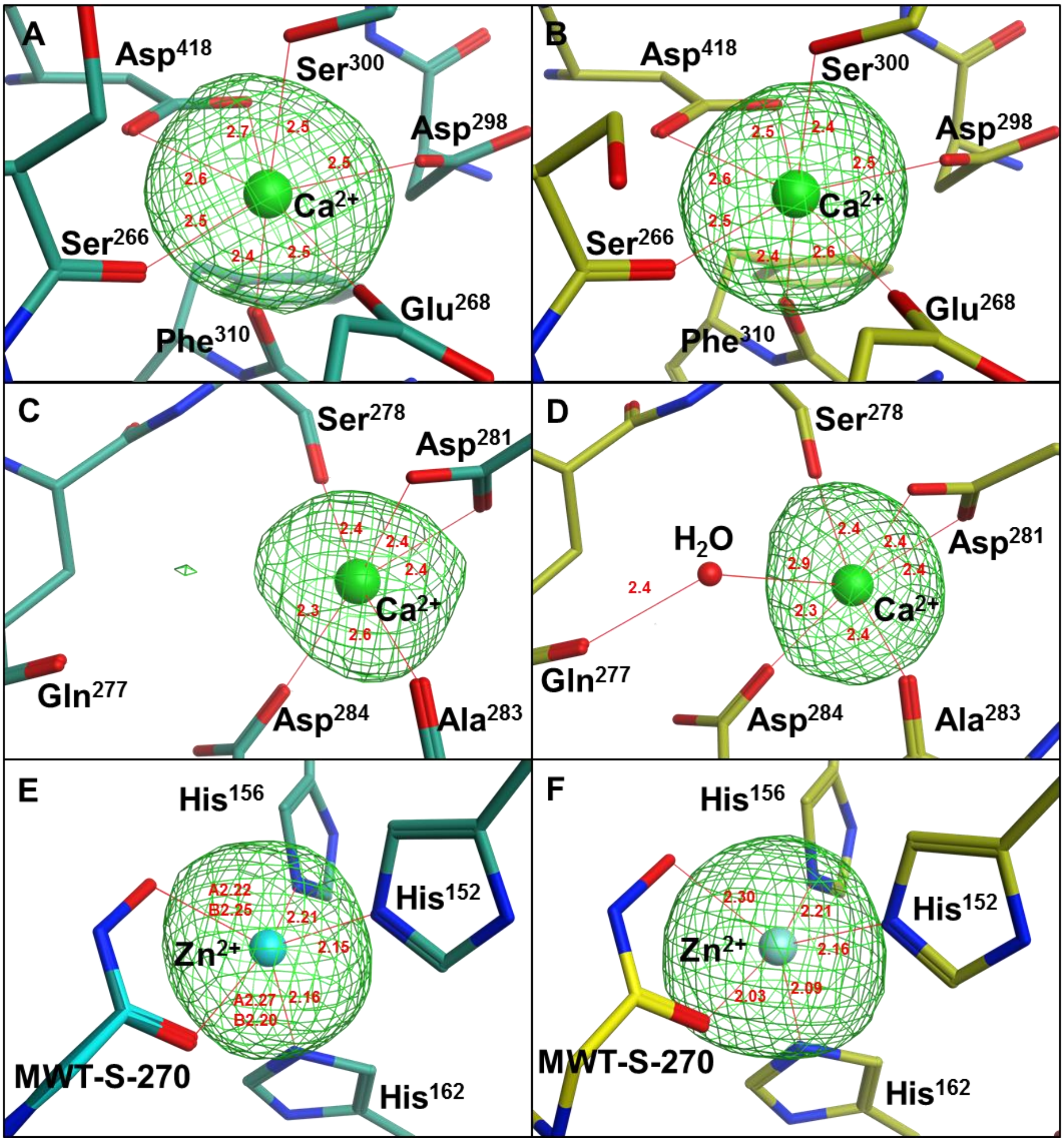

Figure S3: Metal binding sites in Meprin B structure. Six putative metal binding sites occur in the here presented structure of Meprin $\beta^{62\text{-}595}$ (MβΔC) Homodimer (Meprin B), four tentative calcium binding sites in the MAM domain **(A-D)** and two zinc binding sites (**E,F**) within the active site of monomer A and B. Monomer A is schown in green (left) and monomer B in yellow (right), oxygen and nitrogen are colored in red and blue, respectively. Water molecules are represented as small light red balls. Metal binding interactions are indicated as red lines. The binding distances are indicated in Å. An omit map was calculated in the absence of the metal ions. The Fo-Fc difference electron density from these omit map calculation is shown as green mesh, reflecting the presence of the ions. The coordination geometry of zinc is trigonal bipyramidal in monomer A (**E**) and B (**F**). Fo-Fc map is contoured to 5 σ. Tentative calcium ions (green sphere) seems octahedral coordinated (A-D). However, the water binding site in **C** is not resolved. Fo-Fc map is contoured to 3.5 σ, each.

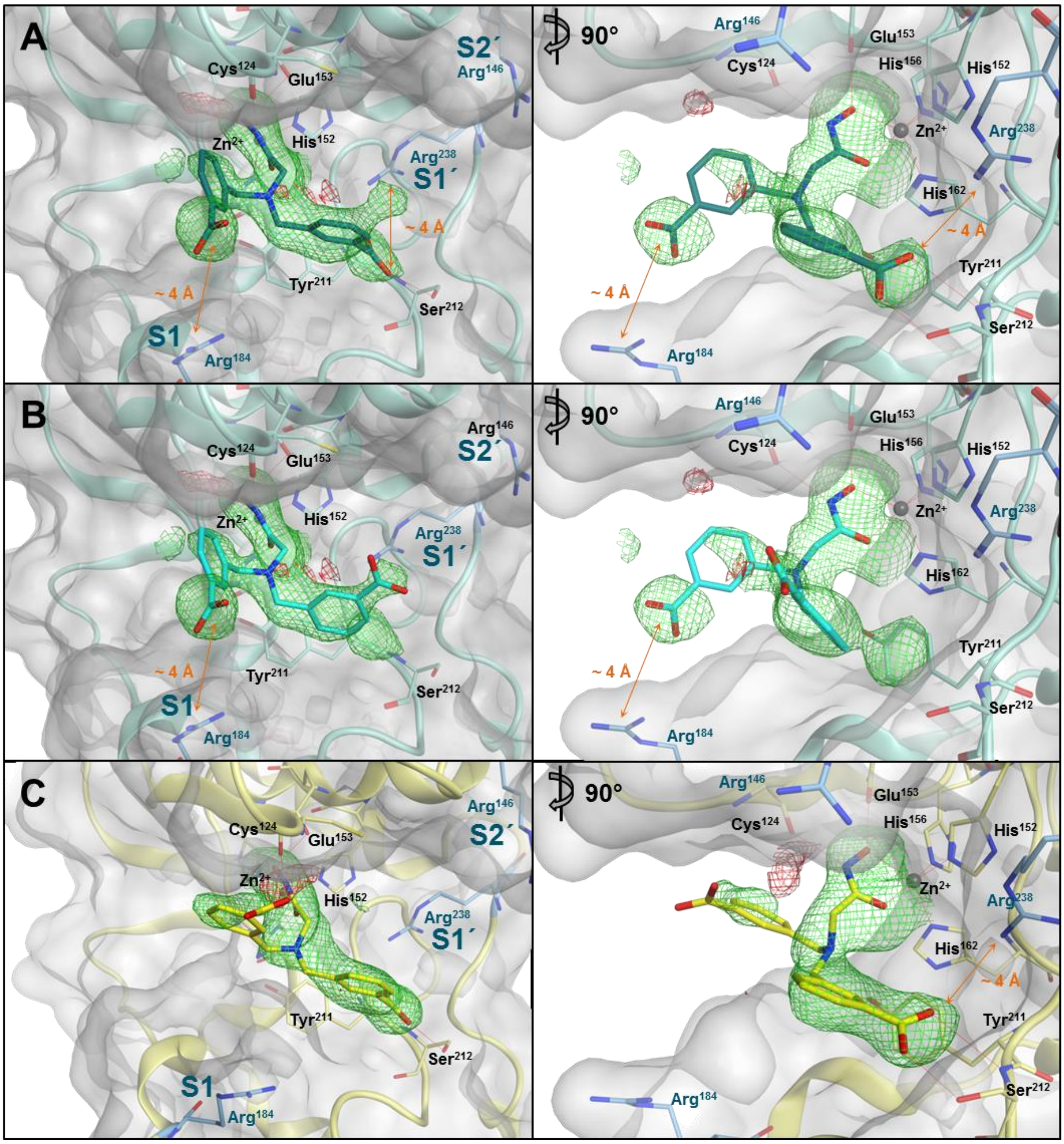

Figure S4: MWT-S-270 bound to the active site of Meprin β. The active site is shown in "standard protease orientation" (left) and turned by 90 ° in side view (right) for monomer A (light green), inhibitor conformation A (dark cyan) (**A**), monomer A, inhibitor conformation B (light cyan) (**B**) and monomer B (light yellow) with built inhibitor (yellow) (**C**). The bound zinc ion is shown as dark grey sphere and the molecular surface in light grey in each panel. Shown is the obtained difference density (Fo-Fc) from an omit map calculation in absence of MWT-S-270, which indicates the presence of the inhibitor. The difference electron density is contoured to 2.5 σ and shown as green mesh. The zinc ion is penta-coordinated by His^{152}, His^{156} and His^{162} and by O1 and O4 of the hydroxamic acid moiety of MWT-S-270. The inhibitor is bound in different conformations in monomer A and B and the difference

density points to higher flexibility in monomer B. Distances between the inhibitor and the respective arginine residues are indicated in orange.

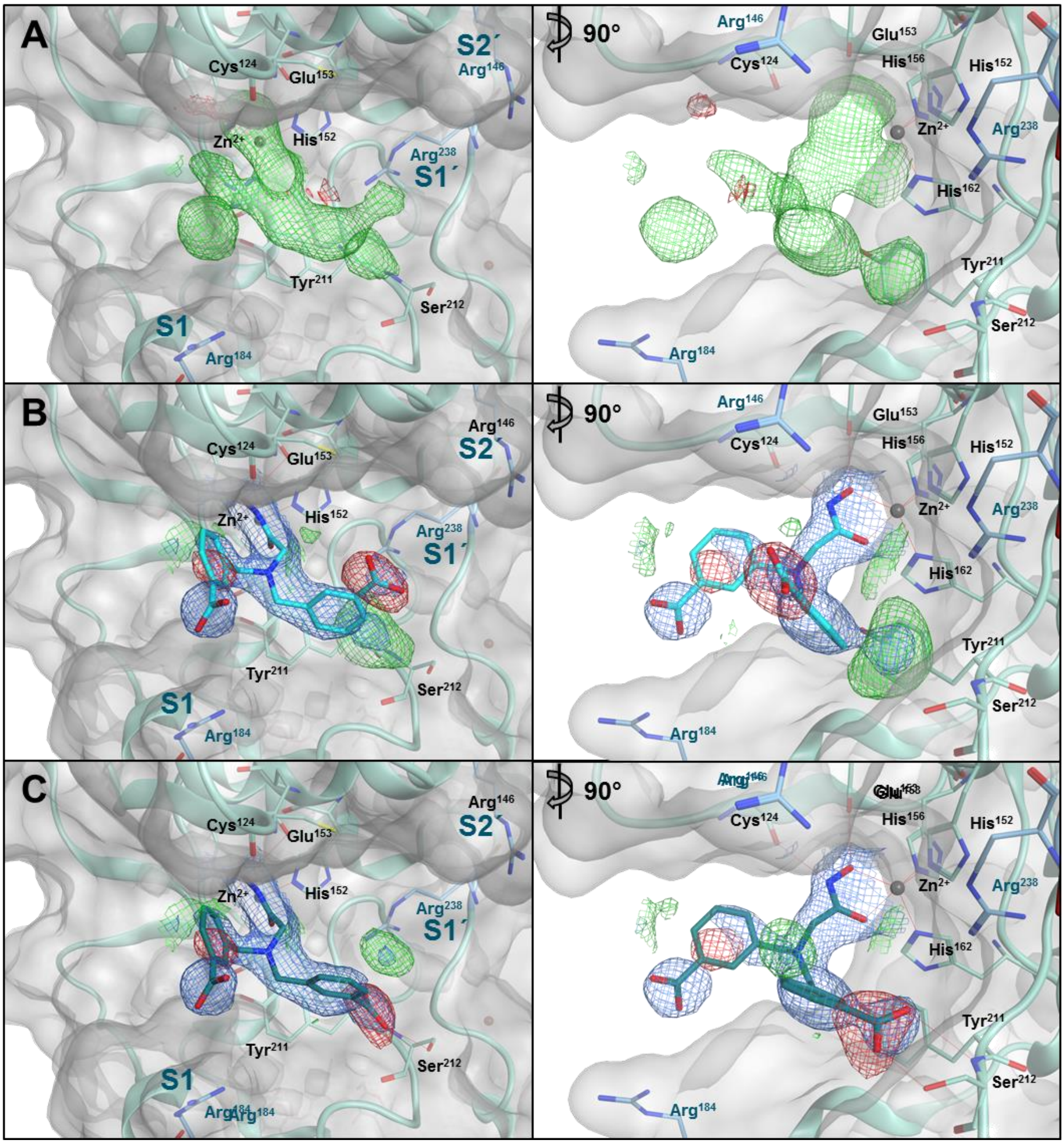

Figure S5: Omit electron density map in the absence of MWT-S-270 in chain A. The active site is shown in "standard protease orientation" (left) and turned by 90 ° in side view (right). Electron density maps were calculated in the absence of the MWT-S-270, both confromers (**A**), in absence of conformer A (**B**) or in absence of conformer B (**C**). 2Fo-Fc electron density (1 σ) is shown as blue mesh. Obtained positive difference electron density (Fo-Fc, 2,5 σ) is shown as green mesh, negative density as red mesh. Positive electron density can be seen at the position of the absent second conformation, negative elctron density at the position of the carboxyl group auf the revealing inhibitor due a lower occupancy in the complex with both inhibitor conformations.

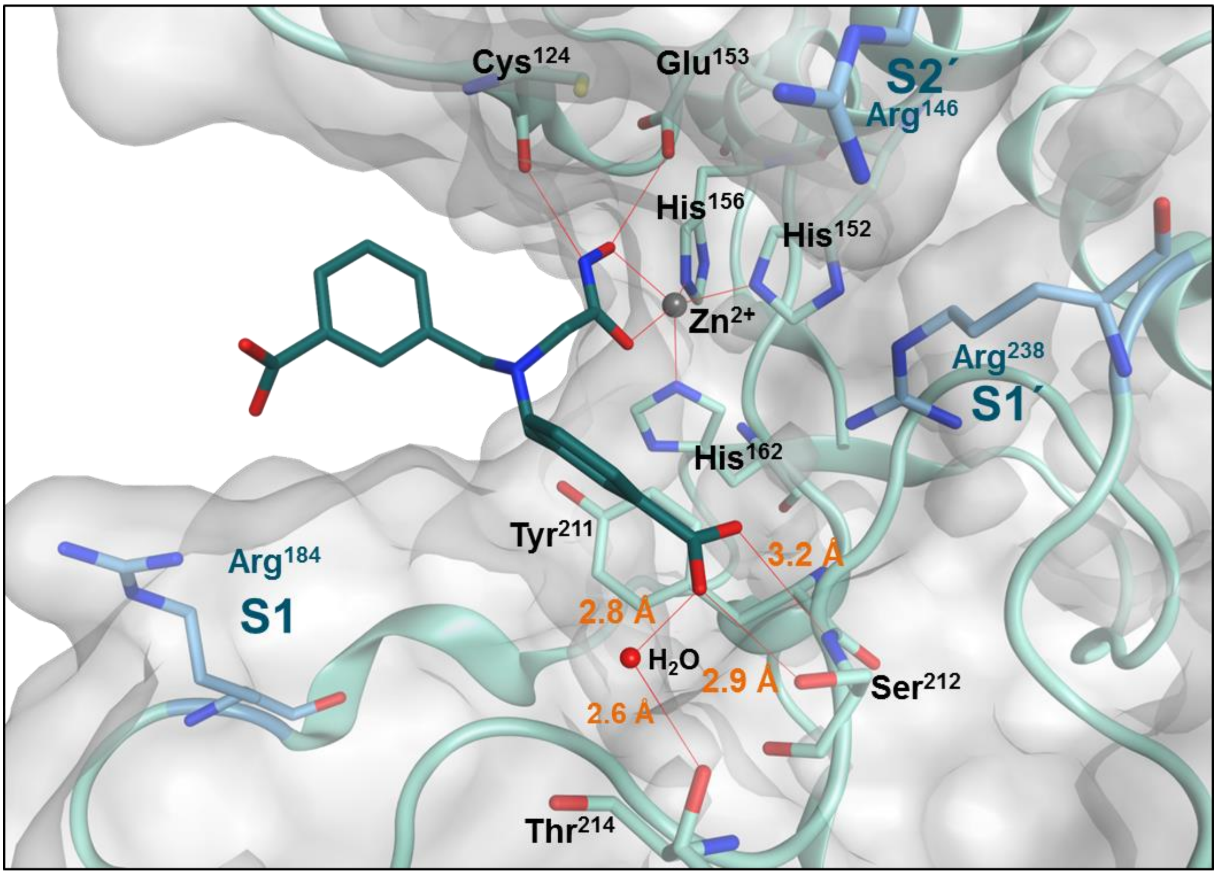

Figure S6: S1´-subsite interactions of Meprin β with MWT-S-270 (shown for monomer A, conformation A). Ribbon and molecular surface are shown in light green. Interactions with the inhibitor MWT-S-270 are shown as red lines, whereby distances are indicated in orange. The carboxylic acid of MWT-S-270 is close to the side chains of Ser^{212} and could interact with Thr^{214} via bridging water molecules.

1. MWT-S-270 (-NaCl)

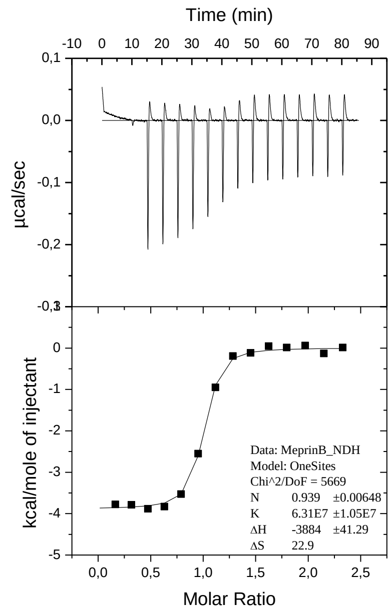

2. MWT-S-270 (+ NaCl)

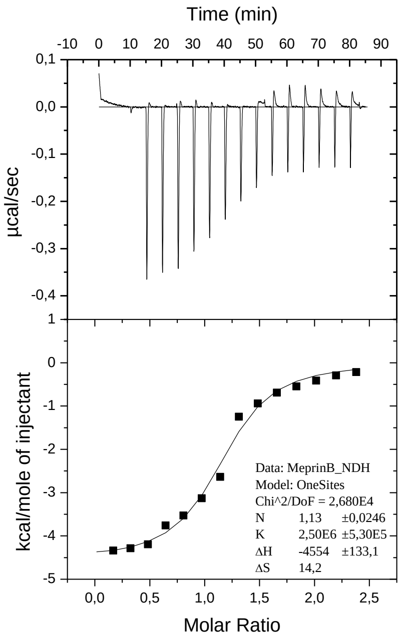

3. MWT-S-416

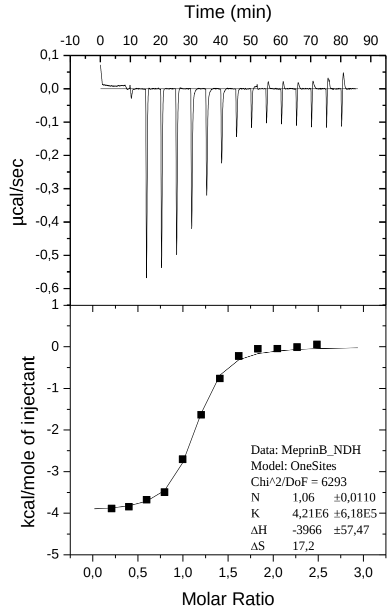

4. MWT-S-396

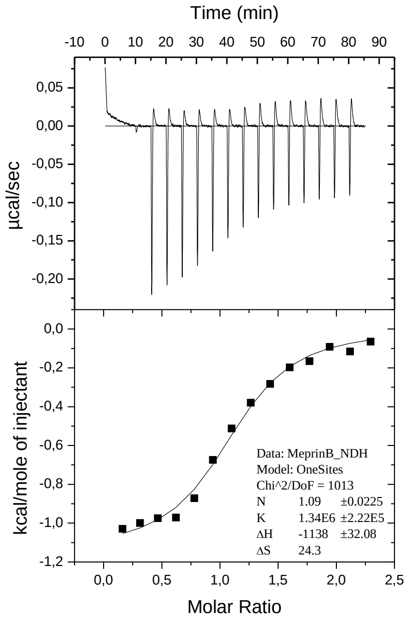

Figure S7: ITC binding curves for the titration of Meprin $\beta^{62\text{-}595}$ (MβΔC) with different inhibitors.

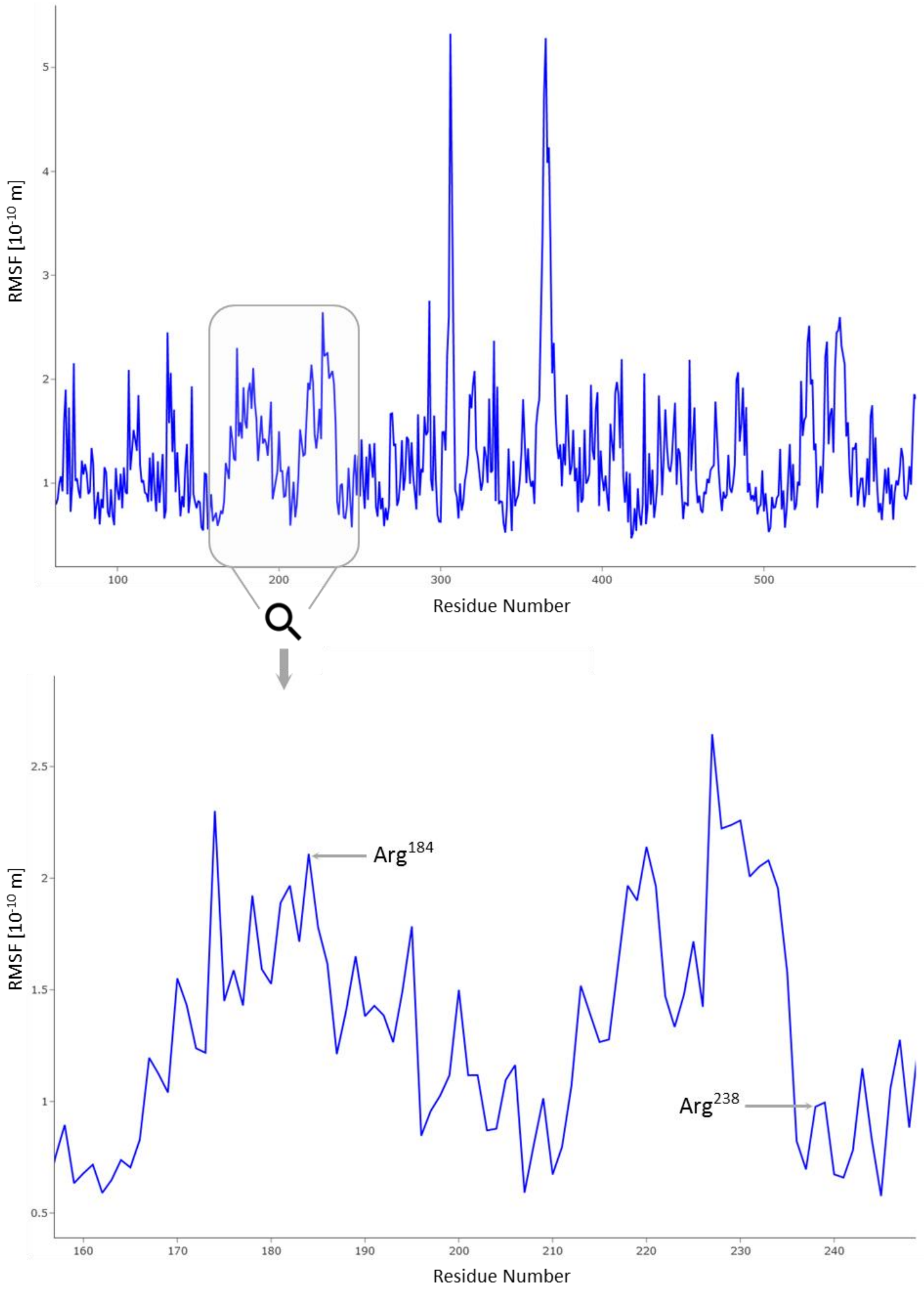

Figure S8: The RMSF of Cα atoms of Meprin β^{62-595}, calculated from a 250-ns MD simulation. The region of S1 and S1′ sub-pockets are magnified at bottom plot.

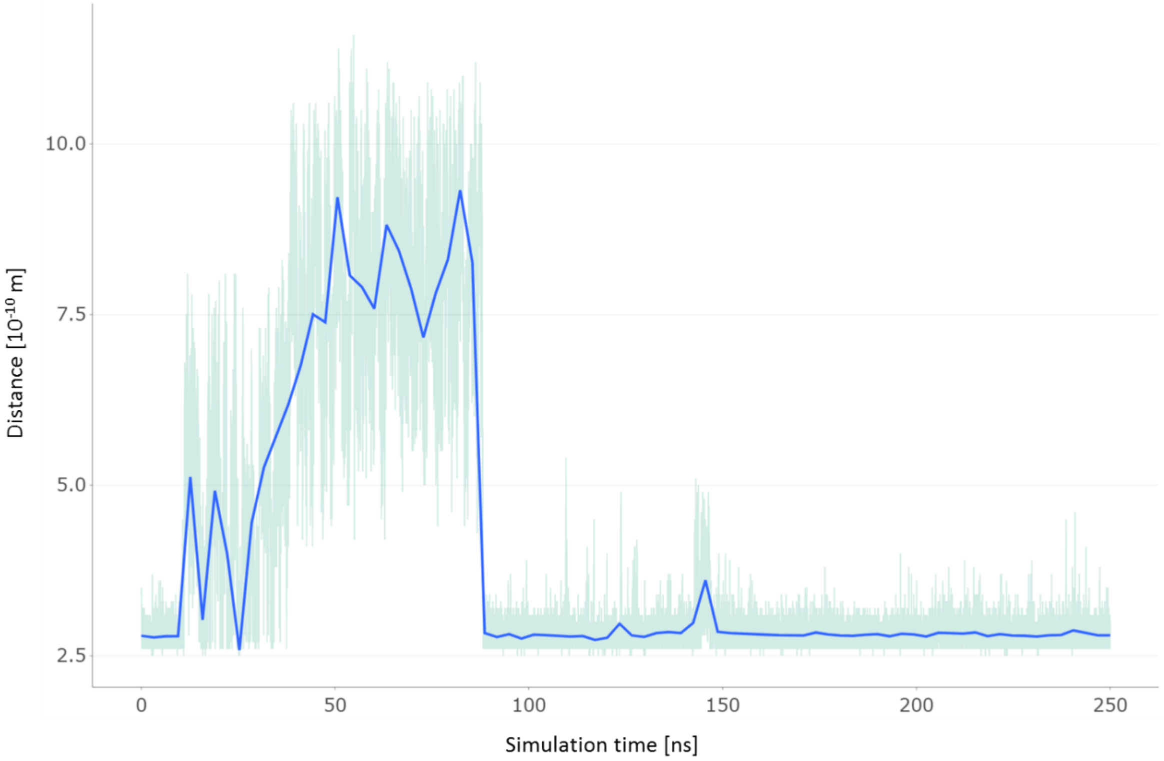

Figure S9: Distance between the guanidine nitrogens of Arg[238] and the interaction partner (O25 & O26) of MWT-S-270 over 250 ns MD. Blue line shows the locally estimated scatterplot smoothing (LOESS).

A

```
                       S1´
Meprin β62-259 NSIIGEKYRWPHTIPYVLEDSLEMNAKGVILNAFERYRLKTCIDFKPWAGETNYISVFKG  121
Meprin α66-263 NGLRDPNTRWTFPIPYILADNLGLNAKGAILYAFEMFRLKSCVDFKPYEGESSYIIFQQF  125
               *.: . : ** . ***:* *.* :****.** *** :***:*:****: **:.** . :

                                       S1´
Meprin β62-259 SGCWSSVGNRRVGKQELSIGANCDRIATVQHEFLHALGFWHEQSRSDRDDYVRIMWDRIL  181
Meprin α66-263 DGCWSEVGDQHVGQ-NISIGQGCAYKAIIEHEILHALGFYHEQSRTDRDDYVNIWWDQIL  184
               .****.**:::**: ::*** .*   * ::**:******:*****:******.* **:**

                 S1                                                        S2´
Meprin β62-259 SGREHNFNTYSDDISDSLNVPYDYTSVMHYSKTAFQN-GTEPTIVTRISDFEDVIGQRMD  240
Meprin α66-263 SGYQHNFDTYDDSLITDLNTPYDYESLMHYQPFSFNKNASVPTITAKIPEFNSIIGQRLD  244
               ** :***:**.*.:  .**.**** *:***.  :*:: .: ***.::* :*:.:****:*

Meprin β62-259 FSDSDLLKLNQLYNCSSSL 259
Meprin α66-263 FSAIDLERLNRMYNCTTTH 263
               **  ** :**::***:::
```

B

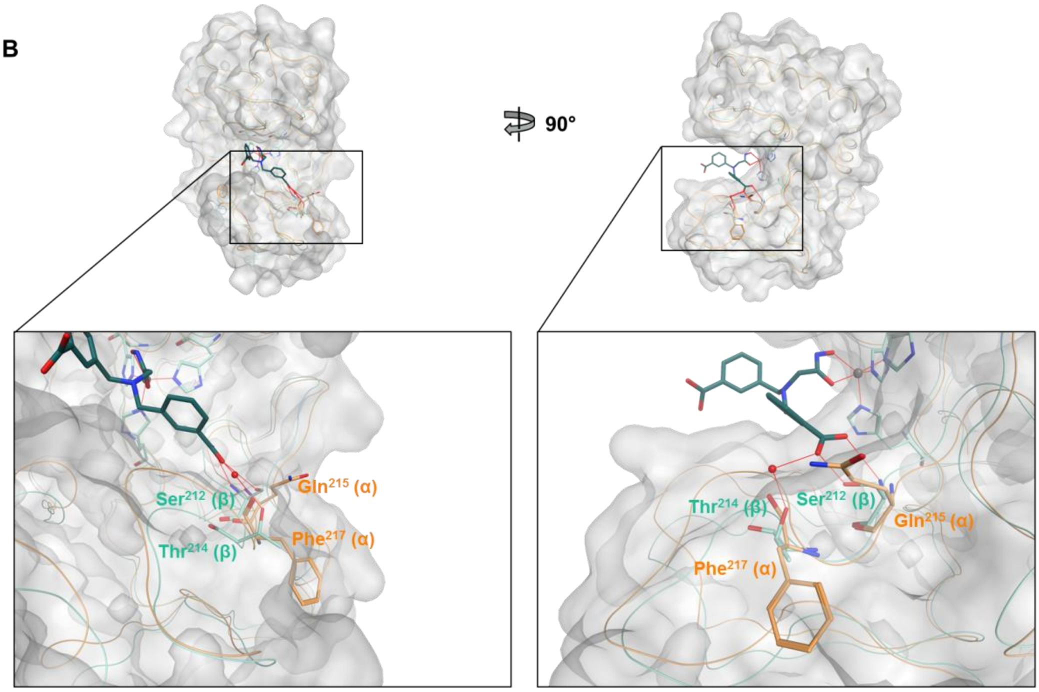

Figure S10: Comparison of the structure of Meprin β with an homology model of Meprin α. A) Sequence alignment of the Meprin β62-259 and Meprin α66-263 protease domains, prepared with Clustal Omega [72]. The arginin residues of subsites S1, S1´and S2 subsite are highlighted in bold. Ser212 and Thr214 are shaded in black. **B)** Superposition of the Meprin β Monomer A (ribbon is shown as line, light green and molecular surface in grey), inhibitor conformation A (dark cyan) with a homology model of Meprin α (ribbon as orange line) [33] in "standart protease orientation" (left) and in "side view" (right). Two of the crucial residues for binding of MWT-S-270 to Meprin β, Ser212 and Thr214, are represented by Gln215 and Phe217 in Meprin α.
